# *‘Every Voice Matters’:* A Photovoice Study on the Personal Impacts of Co‐Production in Recovery Colleges

**DOI:** 10.1111/hex.70441

**Published:** 2025-10-07

**Authors:** Lisa D. Hawke, Shelby McKee, Holly Harris, Amy Hsieh, James Svoboda, Maral Sahaguian, Gail Bellissimo, Melissa Hiebert, Kelly Lawless, George James, Sean Patenaude, Jordana Rovet, Sophie Soklaridis

**Affiliations:** ^1^ University of Toronto Toronto Canada; ^2^ Centre for Addiction and Mental Health Toronto Canada

**Keywords:** co‐production, impacts, lived experience, photovoice

## Abstract

**Background:**

The engagement of people with lived experience (PWLE) of mental health and substance use health challenges in the co‐production of health services, programming and research has many benefits, but how co‐production impacts those involved remains unclear. Recovery Colleges are low‐barrier, generally co‐produced education programmes focused on mental health and wellness. Designed to support individuals on their personal recovery journeys, they provide a meaningful setting to explore the impacts of co‐production.

**Objective:**

This co‐produced study explored the impact of co‐production within recovery‐oriented programming using a photovoice methodology. Photovoice captured the lived experiences and expertise of people involved in Canadian Recovery Colleges as curriculum designers, facilitators and/or students.

**Method:**

A sample of 21 participants with co‐production experience took part in seven photovoice workshops. These culminated in a final photo submission that illustrated how co‐production has impacted them. Eighteen participants completed a focus group discussion on the topic, which was audio recorded, transcribed and analysed using codebook thematic analysis.

**Results:**

Five themes were generated from the data. Participants found that co‐production (1) reduced stigma, (2) provided a space to collectively share lived experience, (3) helped them develop a sense of belonging, (4) helped them advance their personal recovery journeys and (5) supported their personal growth.

**Conclusions:**

This study demonstrated that co‐production in Recovery College settings has a wide range of positive impacts for the individuals involved, across a range of personal factors. The co‐production of services, programme development and research can create positive meaning for those involved in mental health and substance use health settings, as well as potentially other broader health settings, which may aid in their recovery journeys.

**Patient or Public Contribution:**

A Recovery College research subcommittee, including individuals with lived experience of mental health and/or substance use challenges, co‐produced every phase of this study.

## 1 Introduction

There is an ongoing movement towards engaging people with lived experience (PWLE) of mental health and substance use health challenges in the co‐production of health services, programme development and research (i.e., ‘patient engagement’) [1]. PWLE can play instrumental roles in tasks such as setting priorities, conducting data collection or environmental scans, building treatments and services, and translating knowledge [2, 3]. The level of PWLE engagement is often described on a spectrum, taking many forms, including informing, educating, consulting, engaging, co‐designing and co‐producing [4]. Co‐production is the most comprehensive form of engagement that requires the highest degree of power sharing and has potentially the most transformative potential. Acknowledging the intersections between lived and learned expertise, PWLE may bring dual roles as both PWLE and professionals. Lived expertise refers to the expertise gained by experiencing a health condition first hand. Learned expertise refers to knowledge gained through professional or vocational processes. Staff with lived experience can contribute meaningfully to person‐centred service delivery through their identities as both staff members and patients, breaking down power imbalances [5]. Engaging PWLE in co‐production is often described as an ethical imperative and a way to address inequities that can occur in research and clinical practice [6, 7]. Authentic co‐production includes building a team that values the knowledge of PWLE as being equally important to academic knowledge to carry out the work at hand in meaningful ways [8].

A recent literature review identified that when PWLE are engaged in research, there are positive impacts on research quality and various components of the research project, research environment, researchers and research participants, as well as on the PWLE [9]. Impacts on PWLE included positive impacts on mental health and recovery, empowerment, personal and professional growth, and feeling heard and less alone; results also pointed to a transformation of individuals' personal recovery narrative. It has further been demonstrated that engaging PWLE in healthcare service development can improve their satisfaction with their care, as well as health outcomes [3].

Established in the United Kingdom in 2009, Recovery Colleges have become one of the fastest‐growing movements in mental health. There are approximately 220 Recovery Colleges worldwide [10, 11], with over 30 in Canada alone [11]. These low‐barrier mental health and well‐being‐oriented education programmes are co‐produced to support people with mental health or substance use health conditions in their personal recovery journeys [10]. Courses and workshops explore topics ranging from mental health, health and wellness, vocational skills, and life skills to recreation, and creative explorations [12]. Central to the ethos of Recovery Colleges is the notion that services should be produced and delivered by and for PWLE. This leads to a guiding stance of co‐production, a process by which PWLE, people with professional expertise, and those who have multiple perspectives collaborate to design, facilitate and actualise processes, logistics and curricula.

Literature on the impacts of co‐production in Recovery College contexts is only just emerging. The mechanisms of action for student outcomes have been identified [13]. It has been found that Recovery College student outcomes are largely explained by shifting power from medical professionals to PWLE, which enables the formation of different relationships between them and facilitates personal growth. Positive outcomes are thereby seen in the student's life. However, it remains unclear whether engaging in co‐production positively impacts individuals' experiences. Given the importance of co‐production in Recovery College programme development, it is important to understand how the act of co‐producing initiatives affects those who are involved.

### 1.1 Objective

Using a photovoice methodology, this study explored the impact of the co‐production of recovery‐oriented programming on people involved in Canadian Recovery Colleges as lived experience curriculum designers, facilitators or students.

## 2 Methods

Photovoice is a community‐based participatory research methodology that uses photography, expressive imagery, critical thinking and discussion as mechanisms for people to identify, represent and enhance their community [14]. Photovoice developers Wang and Burris [14] identified three main goals of photovoice: (1) to enable people to document and reflect on their community's strengths and concerns; (2) to promote critical dialogue and knowledge about important issues; and (3) to influence policymakers. An additional goal is to influence broader society to facilitate meaningful community change [15]. As a community‐driven approach, photovoice challenges traditional researcher‐centric models of knowledge production [16] by fostering collaborations that centre the lived expertise of marginalised community members and combine it with the professional expertise of researchers. In doing so, photovoice generates valuable insights that can inform policies and services that are relevant to the impacted communities. Co‐production in Recovery College settings, as a complex process with the potential for multifaceted impacts, lends itself to exploration through the photovoice methodology.

The study was co‐produced by the research subcommittee of the local Recovery College, known as the Collaborative Learning College (CLC), which consists of people with lived and learned expertise. Our co‐production processes are reported in Table 1 using a recently developed reporting guideline for reporting on lived experience engagement [17]. We report on the full project as outlined in the Standards for Reporting Qualitative Research (SRQR) checklist [18].

**Table 1:** Description of the lived experience co‐production component of the study.

Domain	Description
Who was involved?	We, the Collaborative Learning College (CLC) Research Subcommittee, are a collaboration of PWLE, Recovery College students and staff, course facilitators, researchers, and members with multiple roles. We came together to share our lived and learned experiences as a group with diversity, yet commonality. Our goal was to collaborate as a group with diverse forms of lived/living experience and academic/professional backgrounds to design and execute research that would benefit the Recovery College movement.
What were the roles, activities and responsibilities?	We co‐designed the research question, the protocol, the photovoice workbook and workshops, the recruitment strategy, the photo gala materials, knowledge translation materials, and other supporting documents. We worked together on developing the codebook, coding the data and selecting quotes. We co‐wrote the manuscript, including this table. We presented briefly on the photovoice project as part of larger presentations to build momentum and enthusiasm.
How did you go about the engagement process?	The engagement process was rooted in the principles of co‐production, honouring diverse experiences and multidirectional learning. We held bi‐weekly virtual meetings, supplemented by email communication, writing and editing virtual documents asynchronously. Meetings began with a comfort agreement to support a harmonious environment that was responsive to individual needs and preferences. Varied levels of engagement were encouraged, such as camera on or off, use of the chat function, and check‐in meetings for members who were unable to attend due to scheduling conflicts. We learned about photovoice from multiple presentations. Decisions were made by consensus when possible, or by majority when necessary, sometimes using voting. Accommodations were provided, including the use of dark‐mode documents. Members who were not employed by CAMH were compensated with an hourly honorarium.
When did the engagement occur?	The CLC Research Subcommittee was formed in 2023 and began with the process of establishing a Recovery College research agenda, which led to the photovoice project. We launched the photovoice process in February 2024 with an informational presentation. Thereafter, meetings occurred online bi‐weekly, with additional meetings where needed. Meetings were typically 2 h in duration, focused on photovoice and alternate topics.
What was the impact?	The co‐production process was carried out throughout the entire project and brought together a variety of members to understand different perspectives, thereby developing a community. Co‐production enhanced the clarity and relevance of the research question, the relevance of the probing questions for participants, and the reporting of the results. Two CLC Research Subcommittee members were also study participants. Co‐production had personal impacts that varied for each member, including academic, professional and personal. It made members feel more empowered, validated and invested in the project as they learned about photovoice, the research process and the co‐production process.

### 2.1 Participants and Recruitment

The photovoice study consisted of 21 participants. To be included, participants had to be involved in a Canadian Recovery College as co‐producers, facilitators or students. We included all three roles to gain a breadth of perspectives about the impacts of various aspects of co‐production. In addition, they had to be aged 16 or older and residing in Canada. The CLC Research Subcommittee co‐produced the recruitment plan and flyer. Recruitment occurred nationally via email and social media, utilising the CAMH institutional X handle and sharing flyers through Canadian Recovery College networks. Potential participants connected with the lived experience Research Analyst (RA) through their preferred communication channels for screening and informed consent. First, participants were screened for eligibility. Those eligible were asked to provide electronically signed consent virtually using WebEx teleconferencing system and the REDCap e‐consent framework in a one‐on‐one meeting [19]. Recruitment was ongoing until the third round of photovoice workshops was launched. Aligning with a previous photovoice project conducted by our team [20], we sought a larger‐than‐average sample to address a current limitation in photovoice research regarding small sample sizes [21]. The study was conducted at a tertiary care research and teaching hospital in an urban centre, and the site of a Canadian Recovery College, the Centre for Addiction and Mental Health (CAMH).

### 2.2 Procedure

All research activities were completed virtually. Consented participants completed a demographic questionnaire on REDCap. All participants were then invited to attend a series of seven 90‐min workshops, which were co‐facilitated by the lived experience RA and a lived experience photographer. Weekly workshops were scheduled based on participant and facilitator availability. Inspired by our previous photovoice project [20], we collectively designed the workshops. Workshop topics included an introduction and discussion of ethics, photography techniques, photo editing, annotating and photography sharing. We also incorporated flexibility into the content, allowing time for participants to express and implement their own ideas. Workshops encouraged reflection and discussion through guiding questions and photography activities that prompted reflection. Examples of prompts throughout the workshops include taking a picture that captures (1) your authentic self, (2) unexpected beauty and (3) working together. These aimed to support participants in capturing a range of photos about topics sometimes abstract in nature that may have some relationship to the experience of co‐production. The final activity was directed by a general probe to take a photograph about the personal impact of co‐production in the Recovery College setting [22].

Participants had the option of turning their cameras on or off and using the chat function to increase accessibility. Extra one‐on‐one sessions were offered to participants who missed a session or required extra support. Workshops took place between 8 October and 19 November 2024 for the first round, between 28 January and 18 March 2025, for the second round, and between 30 January and 20 March 2025, for the third round, with focus groups occurring the week after each round.

Following the workshops, participants were asked to submit final photographs for discussion during the focus groups. They then participated in one of four focus group discussions, each consisting of three to seven participants. The first round of photovoice workshops led to two focus groups (*n* = 3 and 4). The two subsequent rounds had one focus group each (*n* = 4 and 7). Focus groups were co‐facilitated by the RA and a research coordinator. They were audio‐recorded and transcribed automatically by the WebEx system. The RA thoroughly reviewed the transcripts, corrected them for accuracy and de‐identified them.

Participants used personal devices for photography, such as cell phones or cameras. Those without access to a device were provided with a basic digital camera (*n* = 2) for accessibility. Participants received compensation for project activities at a rate decided upon by the CLC Research Subcommittee. Participants received $40 for consent and completing the demographic survey, as well as $60 for each workshop, $80 for the focus group, a $40 one‐time payment for out‐of‐workshop time spent, and $40 for attending the end‐of‐project photo gala. The study was approved by the CAMH Research Ethics Board.

### 2.3 Measures

Inspired by a previous photovoice study conducted by some members of our team [20], the semi‐structured focus group guide and demographic form were co‐designed by the members of the CLC Research Subcommittee. For this study, we reviewed those materials and adapted them. The demographic form captured age, gender, geographical region, ethnicity, education, employment and other basic descriptive variables. Questions in the semi‐structured focus group guide focused on guiding participants to reflect on their photographs and elicit dialogue about the impact of co‐production in Recovery Colleges. The focus group guide is provided in Appendix A. Note that the questions about the experience of the photovoice project were not analysed as part of this study, but are featured in a companion manuscript (in progress).

### 2.4 Data Analysis

Codebook thematic analysis was conducted [23]. This is a standardised qualitative research methodology that begins with developing a codebook based on the findings, then iteratively refining it through coding and discussion. This approach was chosen as it was a strong fit with co‐production as it allows for multiple iterations and refinements through discussion. The RA, research coordinator and lead scientist collaboratively developed a codebook based on discussions of their initial understanding of the transcripts. They built the codebook after the first round of workshops and the first two focus groups. The RA and lead scientist then took the codebook to the CLC Research Subcommittee for discussion and refinement. The RA applied the first edition of the codebook to the transcripts, with openness to new codes. Revisions to the codebook and coding were made, and the third and fourth focus groups were coded into the same codebook, with constant openness to new codes and themes. The CLC Research Subcommittee members collaboratively coded a portion of the data. Tentative codes were discussed at the CLC Research Subcommittee meetings, and themes were generated collaboratively to enhance trustworthiness. The analysis involved multiple cycles through data, from initial coding to theme development, ensuring a coherent representation of participant experiences.

### 2.5 Knowledge Mobilisation

In line with the co‐production orientation of the project, we applied a range of co‐produced creative knowledge mobilisation activities to this project. Notably, we hosted a 90‐min virtual photo gala with approximately 35 attendees, including 10 participants who had the opportunity to present and answer questions about their work to community members. We shared the photo gala invitation across Canadian Recovery Colleges for a wide reach. From the photo gala, we created an engaging YouTube video highlighting some of the findings [24], including the photos and narration of some participants. We also created an institutional webpage presenting the photos and annotations from the project [25]. We created a photobook that was sent to all participants, the co‐production group, and people involved in the project. We continue to explore other co‐produced knowledge translation activities, such as social media and institutional opportunities.

### 2.6 Positionality

The CLC Research Subcommittee is comprised of a group of people with lived expertise and learned expertise, as well as a blend of both perspectives. Members conduct co‐production on a regular basis and see its positive impacts first hand. They discussed their perspectives on co‐production, including the potential of a bias towards positive impacts, and made efforts to remain open to all impacts in their work together.

## 3 Results

We had a total of 21 participants complete at least one workshop. Nineteen completed all seven workshops or attended a makeup session in lieu of a workshop, and 18 completed the focus group. In addition, eight participants requested and attended an extra meeting with the RA and the lived experience photographer for additional support. Participant age was 50.7 on average (SD = 12.7, range = 32–74, 4 missing). Sixteen (76.2%) of 21 participants had experience co‐producing content in Recovery College settings, and 18 (85.7%) had experience as Recovery College students. Given the overlap between facilitator and student roles, we did not distinguish between them in the results. Participant characteristics are presented in Table 2. While the majority of participants were women, were born in Canada and spoke English as a first language, other profiles were represented. There was diversity across other demographic characteristics.

**Table 2:** Demographic characteristics of the *N* = 21 photovoice participants.

Demographic characteristic		*n* (%)
Gender	Man	2 (9.5)
	Woman	16 (76.2)
	Transgender/non‐binary	3 (14.3)
Born in Canada	Yes	18 (85.7)
First language English	Yes	19 (90.5)
Ethnic/cultural background	White	10 (47.6)
	Indigenous Peoples	4 (19.0)
	East Asian	2 (9.5)
	South Asian	2 (9.5)
	Middle Eastern	1 (4.8)
	Multiple backgrounds	2 (9.5)
Employed	Yes	14 (66.7)
Receiving financial support	Yes	8 (38.1)
Education	High school or less	2 (9.5)
	Some post‐secondary	4 (19.0)
	Post‐secondary degree/diploma/certificate	15 (71.4)

Five themes were generated from the data: (1) Reducing stigma; (2) A space to collectively share lived experience; (3) A sense of belonging; (4) Fostering well‐being and recovery and (5) Experiencing personal growth. The themes and sub‐themes are summarised in Table 3 and described below, with representative quotes.

**Table 3:** Summary of themes and sub‐themes generated from the data.

Theme	Sub‐theme
Reducing stigma	Non‐judgemental space
	Authenticity
A space to collectively share lived experience	Hearing lived experience
	Sharing lived experience
A sense of belonging	Being accepted by others
	Being connected to others
Fostering well‐being and recovery	Rediscovering voice
	Embracing the recovery journey
	Shifting perspectives
Experiencing personal growth	Mindfulness
	Awareness of self and others
	Rebuild confidence
	Finding purpose and meaning
	Sense of accomplishment

### 3.1 Reducing Stigma

Participants described the co‐production environment of Recovery Colleges as an opportunity to reduce stigma via a non‐judgemental space and authenticity. By sharing experiences, participants found opportunities to confront internalised stigma and rewrite narratives shaped by shame or marginalisation. As one lived experience facilitator explained, ‘*[W]e talk a lot about stigma—I myself know what it feels like to be stigmatized (…). Co‐production is so important—you can't judge a book by its cover’ (Participant 1).*

Creating non‐judgemental spaces was seen as a key way that co‐production helped reduce stigma. Although many participants described initial hesitation to share their experiences, the witnessing of others express emotions often considered taboo—such as anger, shame or grief—created a non‐judgemental space that helped normalise those feelings:

I just felt like everyone like could relax their body and just be like, oh yeah, I can say what I need to say here and I can talk about it—and not worry about being judged, of being misunderstood.

Participant 2

Others described a sense of comfort in settings where co‐production takes place: *‘[Y]ou're around people who get it, so there's NO shame—you can develop a comfort zone’ (Participant 3).* Another participant reflected:

[B]ringing up subjects that are sometimes touchy, in a way that is very healthy and full of life (…) makes the person think, “well, maybe I should do more of that—maybe I can do more of that.”

Participant 4

This non‐judgemental atmosphere encouraged self‐expression, honesty and mutual understanding.

**Figure 1:**
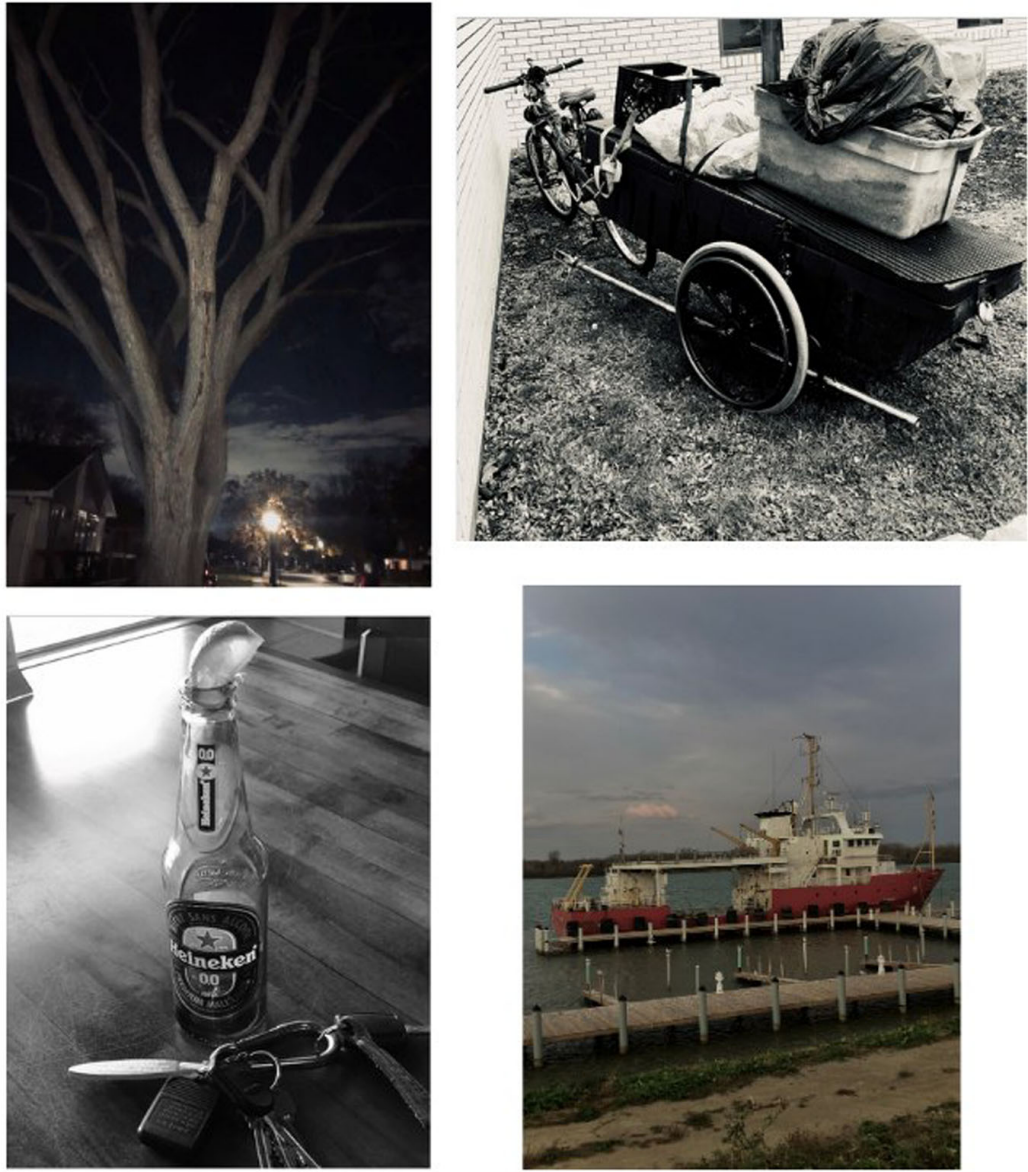
Participant photograph and annotation. ‘My photos and narrative centre around a personal journey of recovery, transformation, and resilience—marked by both emotional vulnerability and profound growth. The photos reflect various aspects of this path, from symbols of sobriety and past struggles to moments of joy, peace, and reconnection with purpose. Throughout these photographs, the focus remains on reducing stigma, celebrating small wins, and creating inclusive, supportive spaces for all—especially those with lived and living experience. Whether it's a non‐alcoholic beer as a symbol of progress or a photo that challenges public assumptions about mental illness, the message is clear: authenticity, empathy, and co‐produced/peer‐led support have the power to save lives and build stronger communities’.

Authenticity was also a central strategy for reducing stigma. Participants—both students and facilitators—emphasised the importance of being able to ‘*come as ourselves’ (Participant 5)* and reclaim aspects of identity that had once being sources of shame. As one participant put it, ‘*its okay to be myself—my kind of crazy self’ (Participant 6).* This reclamation helped reframe historically stigmatised identities as sources of strength and connection. See Figure 1 for an illustration of how the environment of authenticity reduced stigma.

However, some participants acknowledged that stigma still surfaced—even in co‐produced spaces. Comments from past facilitators and others occasionally reflected lingering bias, such as assumptions about participants' expressions or abilities. ‘*Why do you gotta be so blah all the time?’* one participant recalled being asked, before responding ‘*Cause I am’ (Participant 7).*

By fostering non‐judgemental, authentic environments that reduce stigma, co‐production created the necessary foundation for participants to safely share their lived/living experiences and listen to others.

### 3.2 Space to Collectively Share Lived Experience

Participants discussed the power of co‐production in providing a space to share one's own lived experience and to hear the lived experience of others. Being in a co‐produced environment had a large impact on some participants, with one stating, ‘*The co‐production is kind of like the magic of it’ (Participant 2)*. Another participant highlighted the value of mutual storytelling: ‘*When it comes to co‐production, what part I think was meaningful was being able to hear people's experiences’ (Participant 8).* These collective environments fostered emotional resonance and mutual learning, as one facilitator shared:

That's what I really like about Recovery Colleges too, because you gain the wisdom of the group—I learned so much more sometimes from the participants in my classes than I feel like I'm teaching them, and that's why they're so powerful.

Participant 9

Recovery College co‐production settings provided opportunities to express vulnerability, which can be a transformative process. For some, hearing others' stories was an emotional turning point:

I hate crying but I sobbed my whole way through that group—20 years into my journey to actually discover that there's so many people who have their same or similar stories.

Participant 7

These reflections point to how Recovery Colleges are co‐constructed, with participants and facilitators contributing to collective insight and knowledge exchange through shared experiences.

A co‐produced space within a Recovery College gave individuals the opportunity to share their own experiences. These moments affirmed the healing potential of shared experience within a co‐produced space:

I think that we're all human at the end of the day and everybody has mental health. We can all work on our mental health and we can do that by sharing our experiences by not being alone.

Participant 10

Participants also described how expressing their own experiences led to self‐discovery:

Just the experience of sharing myself. I discover a flower within me and it's unexpected—sometimes, you know, the unexpected beauty.

Participant 11

**Figure 2:**
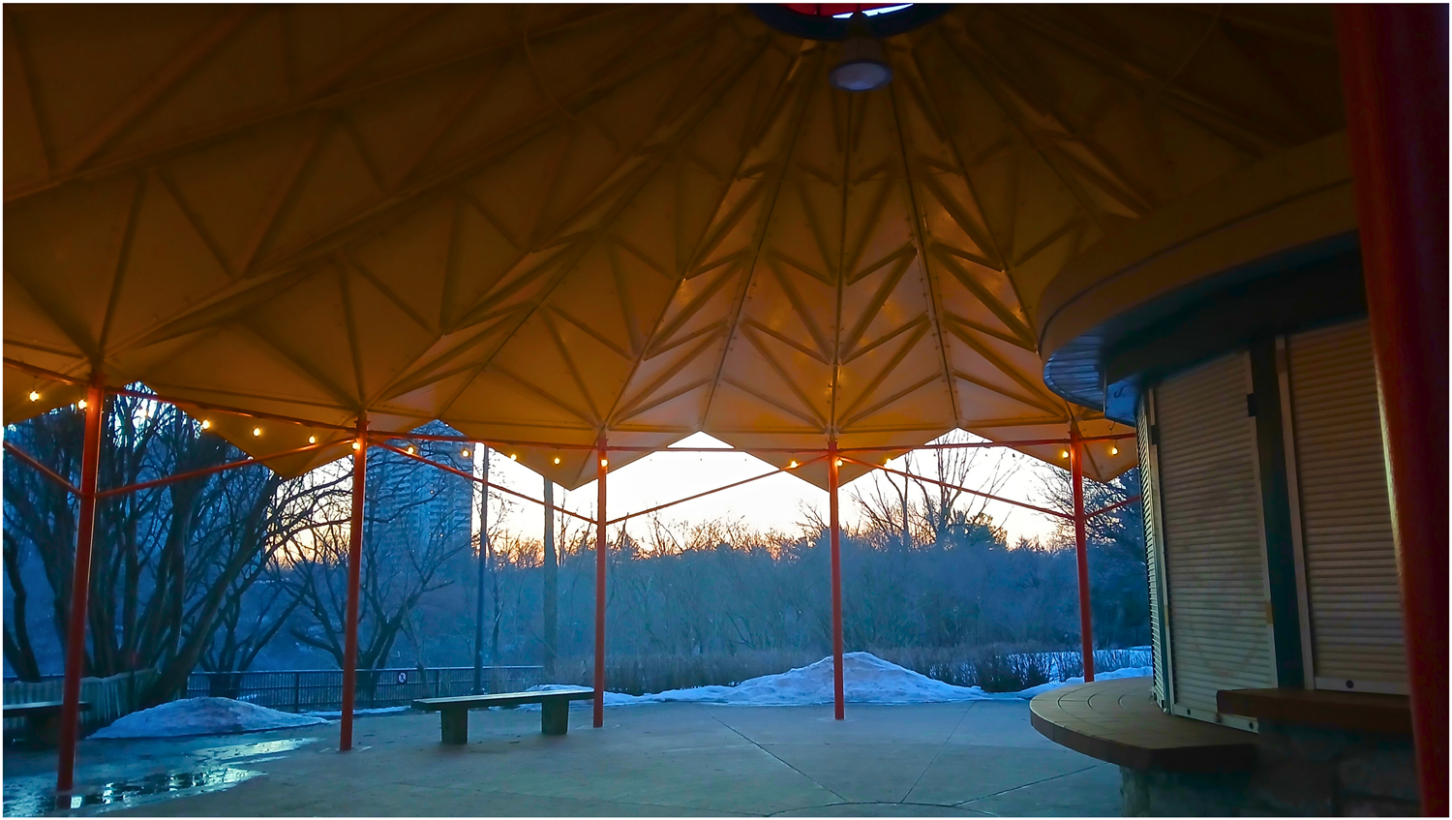
Participant photograph and annotation. ‘Under the Hen's Wing**:** The yellow metal roof of the shelter reminds me of a mother hen's wing—offering protection, warmth, and comfort. It reflects my experience of being supported at the CLC, especially during difficult and vulnerable times. In that sheltering space, I've felt held—able to be where I am, in terms of both ability and recovery. Because of my disability, I've often encountered barriers in professional spaces. At the CLC, I've felt genuinely supported to show up as I am. The culture of flexibility, understanding, and non‐judgment has allowed me to stay engaged, contribute meaningfully, and feel supported. It's rare to find a space that not only accommodates individual needs, but also values each person's unique ways of contributing’.

See Figure 2, where this participant further describes the unexpected beauty of self‐discovery that co‐produced spaces can offer through a space to share lived experience.

However, the impact of sharing experiences depended heavily on facilitation and group dynamics. Some participants highlighted the emotional risk of disclosure and the importance of trust: ‘*You have some kind of trust in others that we wouldn't—belittle what you've done’ (Participant 12).* Others reflected on the need for facilitators who participants can relate to, ‘*someone they can see aspects of themselves in’ (Participant 9),* and the importance of emotional balance when engaging with complex topics: ‘*That balance really needs to be there’ (Participant 2).*

These acts of storytelling and mutual listening did not just encourage self‐discovery, but also laid the foundation for a deeper sense of connection, acceptance and belonging within co‐produced Recovery College spaces.

### 3.3 A Sense of Belonging

**Figure 3:**
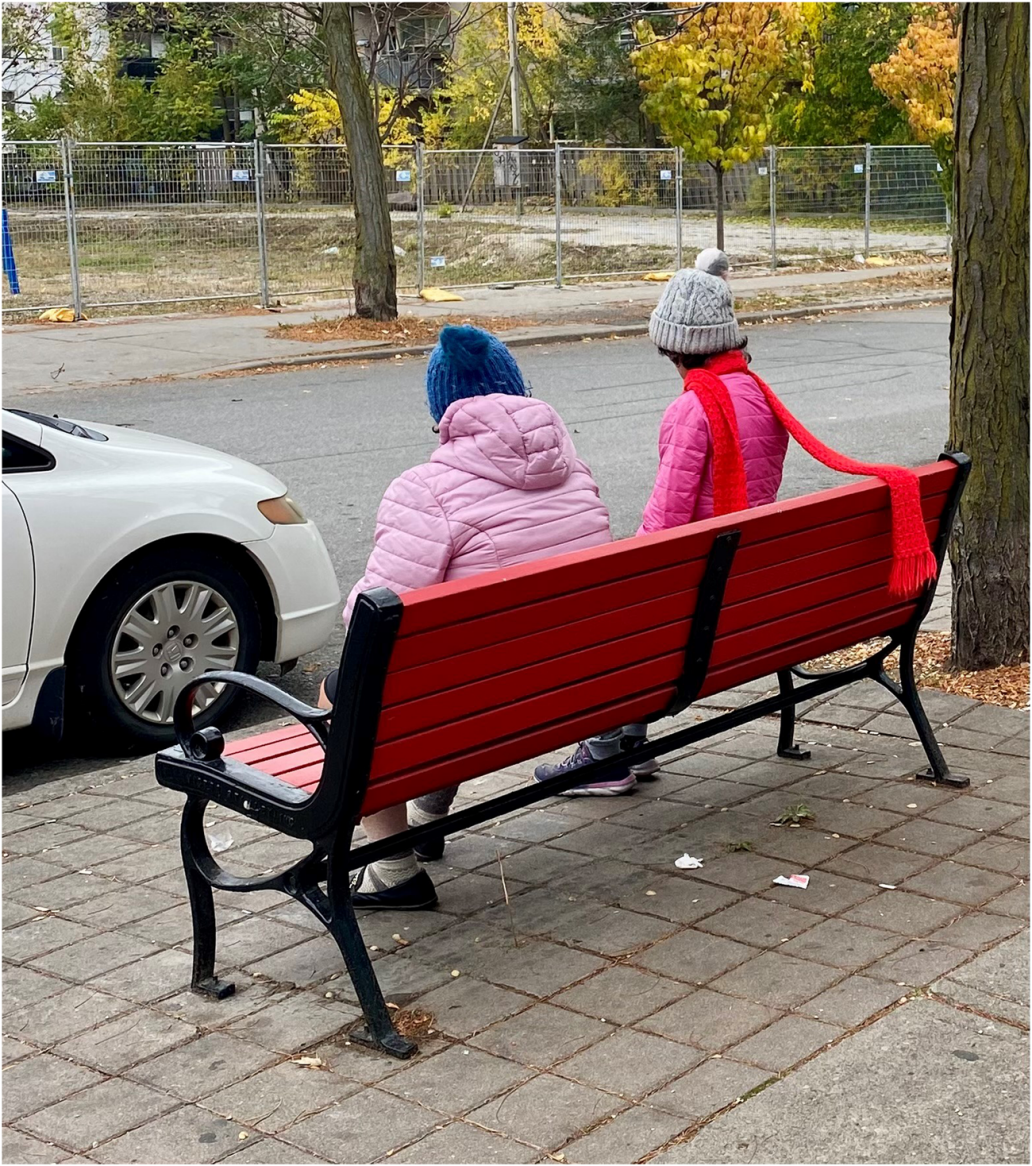
Participant photograph and annotation. ‘In a few short weeks in a Recovery College setting, we can build community and combat our feelings of despair and isolation as it relates to our mental illness, our addictions, our grief, and our loneliness. In this photo and this poem I was trying to capture the idea that we can conquer these feelings of disconnection and isolation by reaching out our hands—one to ask for help when we need it, the other to offer support when we can’. *We are all destined to die, so let me ask you this: Did you share a bench, gift a scarf, hold on to someone's grief? Did you worship where you rested, on pinecone, sun and rain? Did you wipe a tear, kiss a cheek, keep a tender heart from harm? Did you love enough? And let me ask you this as well: When you suffered and despaired, did you remember, yes, remember that you were not alone*.

Participants discussed the sense of belonging through the experience of being accepted and connected with other individuals with lived and living experience in the co‐produced space. As one participant reflected, ‘*as people, we sort of need to belong to a social group and feel like our contributions are being valued (…) it sort of did increase my sense of belonging’ (Participant 13).* This sense of social connection was echoed by another who noted the ‘*incredible importance of social connection*’ *(Participant 2),* where co‐produced spaces can help combat feelings of despair and isolation. See Figure 3, where this participant further illustrates how these environments foster genuine connection and belonging. These reflections reveal how belonging is often built through the meaningful connections formed in co‐produced Recovery College environments.

Participants described a strong sense of belonging that stemmed from feeling accepted by others for who they are, without judgement or the need to conform. One participant described the co‐produced Recovery College space as one where ‘*learning, sharing, growing, and allowing people to be accepted’* created ‘*magical transformations that changed everything’ (Participant 4).* For many, the co‐produced spaces offered a rare environment where people with lived and living experience could ‘*get to the point where we feel like we really belong in our classes, in our groups, in—however we participate in Recovery Colleges’ (Participant 7).* This sense of unconditional acceptance allowed participants to show up authentically, fostering deeper connection, confidence and a true feeling of belonging.

Participants emphasised how meaningful connections to others helped foster a sense of belonging. One participant highlighted the importance of co‐produced organisations that create opportunities for connection and healing: ‘*There is power in connection and community’* and ‘*It really helped me see how beautiful it is to kind of sometimes connect with strangers in different ways’ (Participant 8*). Other participants stated that throughout their years in co‐produced Recovery College spaces, what stuck out to them was ‘*that connection piece’ (Participant 10)* and how they made ‘*incredible friendships [and] got to know some amazing people’ (Participant 14).* These reflections illustrate how shared experiences and authentic relationships within co‐produced Recovery Colleges were key to building a lasting sense of belonging through acceptance and connection.

**Figure 4:**
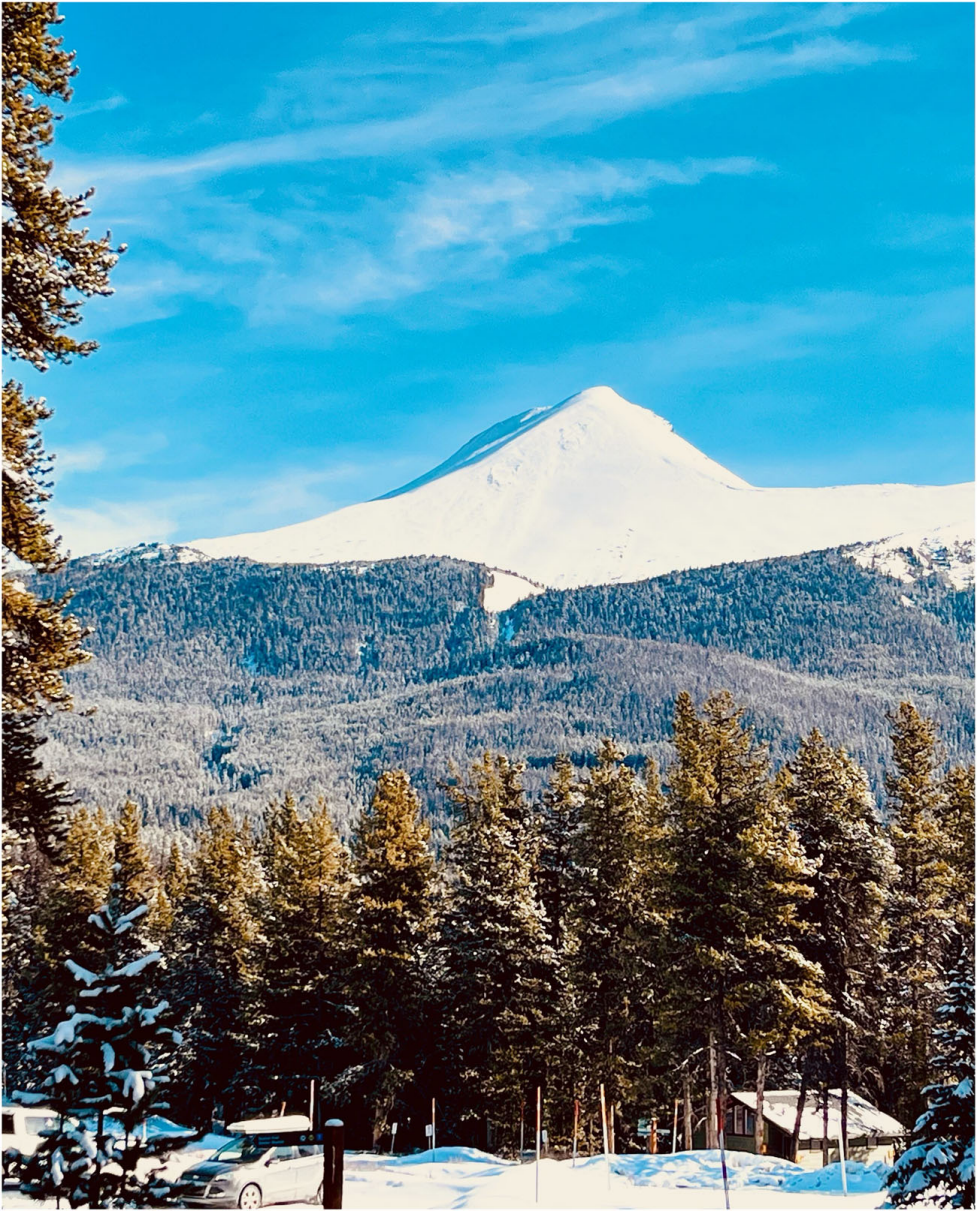
Participant photograph and annotation. ‘We talked about rediscovering our hobbies and things we enjoyed before our health challenges in new ways. I had always loved the mountains. Hiking and camping, even backpacking, but once I became chronically ill I was no longer able to do those things and I gave up on the mountains. Through this project I was able to rediscover my love of the mountains by staying in a hotel and going and sitting at various lookouts. I can still have a vacation in the mountains and find joy in it!’.

This sense of belonging provided a critical stepping stone towards healing by supporting participants as they began to reconnect with themselves, their recovery journey and their overall well‐being (Figure 4).

### 3.4 Fostering Personal Well‐Being and Recovery

Co‐produced programmes in Recovery College settings fostered well‐being and recovery by supporting individuals in reconnecting with their voice, reflecting on their journey and shifting their perspectives. Rediscovering, strengthening and expressing voice was central to their recovery process. Participants shared how co‐produced Recovery Colleges offered the opportunity to reclaim parts of themselves that had been diminished by past challenges. One participant described ‘*the journey to rediscovering one's own voice’* (*Participant 8)* while another emphasised,

I felt like I didn't have a voice—and doing all this stuff has just helped redevelop all that and helps me find my integrity again.

Participant 9

Voice was also seen as a collective act, with one participant asserting ‘*co‐production is (…) giving the people that [have] been through it or in the thick of it, a voice (…) nothing about us without us’ (Participant 5).* At the same time, participants acknowledged challenges, cautioning that ‘*some people's voices can get lost (…) some people's voices are louder’* (*Participant 10),* calling attention to the importance of intentional facilitation to ensure that all voices are heard and valued.

**Figure 5:**
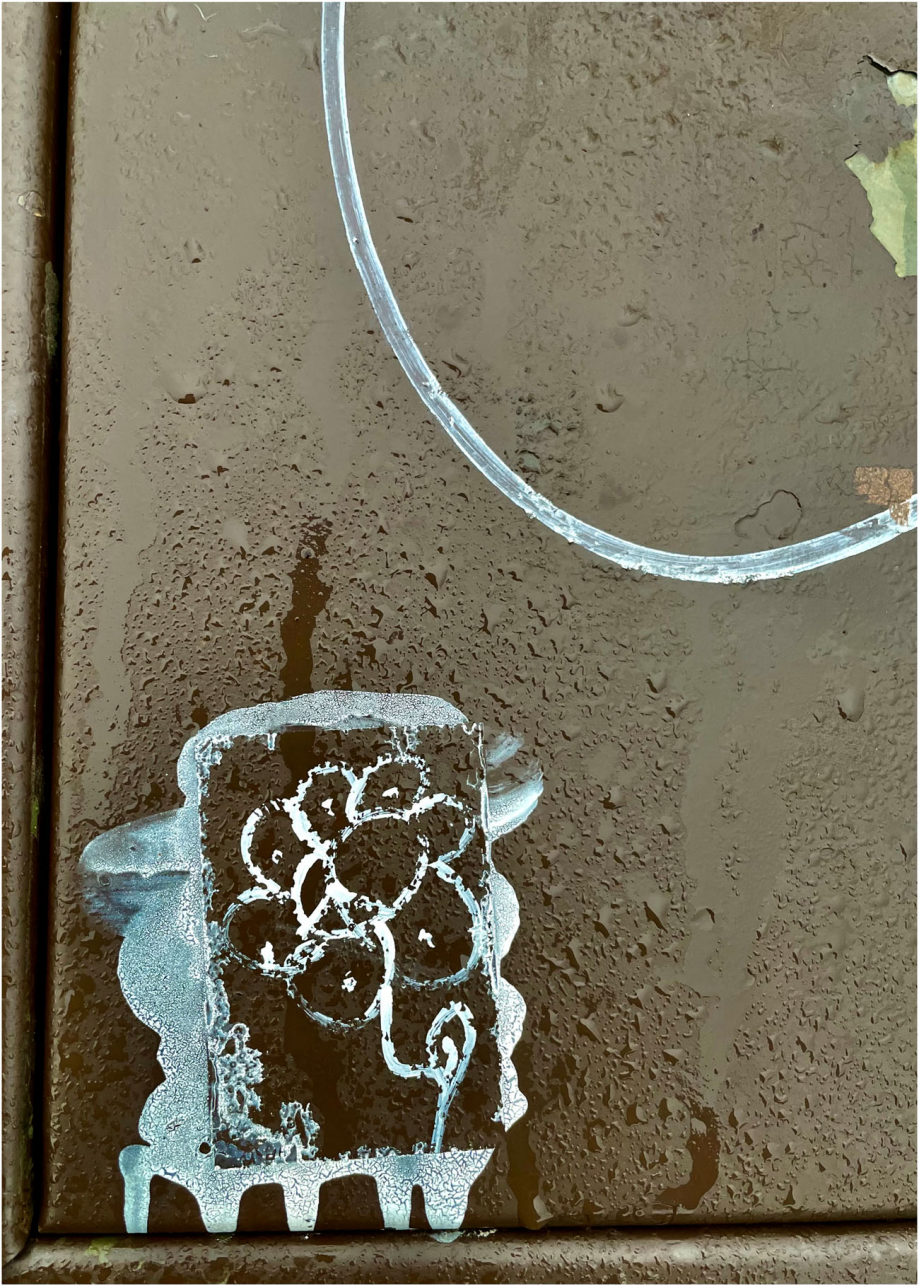
Participant photograph and annotation. ‘Nothing works when forced. Only when the conditions are right will a flower blossom from a seed. That seed receives so much support and love, the perfect amount of water, the perfect amount of light. The process of finding peace takes time—just like how all flowers were once just a seed in mud. The peace we find within is often supported by those outside ourselves, just like the clouds provide rain and the sun provides warmth’.

Recovery Colleges offered a co‐produced environment that supported personal well‐being by enabling them to embrace their recovery journey. These co‐produced spaces were often described as therapeutic, offering safety, encouragement and healing on one's own terms. As one participant shared, ‘*I feel like it's also like it's therapy in a way when you go to Recovery College’ (Participant 15).* Participants described how these spaces allowed them to unfold and grow gradually like ‘*tight little stressed out buds’* opening into ‘*full freaking bloom’* (*Participant 7).* As one facilitator explained, it is ‘*being able to work with people to help them realize that like you know life doesn't end when you get that diagnosis’ (Participant 5).* The recovery journey was also viewed as a non‐linear process made possible through understanding and peer support. One participant emphasised the importance of ‘*walking the journey together as people with lived experience’* (*Participant 1).* Another described recovery as ‘*moving forward—from winter to spring’ (Participant 3).* These reflections highlight how co‐production can promote well‐being and recovery while requiring care and intention to ensure all voices are uplifted along their journey. Figure 5 captures that same forward movement—rediscovering joy in accessible ways as a metaphor for reframed recovery.

Finally, Recovery Colleges supported participants in shifting their perspectives of themselves, their challenges and what was possible in their recovery. As one participant reflected, ‘*I don't even think I realized I had a mental health journey until I started at Recovery Colleges’* (*Participant 10)*, underscoring how these co‐produced spaces helped make recovery more visible and tangible. Participants also highlighted how the process encouraged introspection and reframing adversity, noting how co‐production helped them:

recognize the need for reflection and solitude, and change my perspective on how I'm responding to the change and the things that are happening in my life.

Participant 8

These insights show how co‐production can help individuals reframe limitations, find meaning in new ways and approach recovery with greater emotional clarity. As participants continued their recovery journeys, many described experiencing a renewed sense of voice and personal growth, as well as a shifted perspective, which were cultivated through co‐produced spaces that supported reflection, confidence and the discovery of value, purpose and accomplishment.

### 3.5 Experiencing Personal Growth

Participants described experiencing personal growth in the co‐produced setting as a gradual, individualised process that included mindfulness, awareness of self and others, confidence, a sense of purpose and value, as well as a sense of accomplishment. As one participant shared:

I wasn't quite blossoming when I made it to recovery colleges, but I think I was on my way (…) Recovery college gave us all space to figure our stuff out and to grow.

Participant 9

See Figure 5, where this participant illustrates how a flower parallels their journey from vulnerability to flourishing in relation to the co‐produced space of Recovery Colleges.

Others described the co‐produced spaces as ‘*nurturing’ (Participant 11)* and emphasised that ‘*there's a need for growth that comes with—that process’ (Participant 8).* Together, these reflections highlight how Recovery Colleges provided the supportive conditions needed for personal growth to take root and unfold in personally meaningful ways.

Specifically, co‐production in Recovery Colleges nurtured personal growth by encouraging mindfulness and emotional presence. One participant noted becoming ‘*more patient, more listening and more relaxed’* and learning that ‘*you can share if you want to share’ (Participant 12)*. Another said, ‘*just allowing all those emotions to come through’ (Participant 2)* was a key discovery. These reflections show how co‐production spaces supported greater mindful awareness as part of the personal growth process.

These co‐produced spaces also encouraged awareness of self and others in ways that deepened understanding. Participants described gaining awareness and insight into their behaviours and communication with others. One shared how they learned to better listen and reduce disruption in group settings, especially as someone with autism:

I was teaching myself—to make the sessions not be as disruptive—and really listening—and appreciating [when] people share.

Participant 12

Others described personal insight emerging through challenges: ‘*Recovery College has really taught me about my own self‐awareness and my own mental health (…) discovering more of myself every day’ (Participant 10).* These reflections illustrate how co‐production not only fostered individual growth, but also strengthened participants' ability to navigate shared spaces with greater empathy, intention and awareness.

Recovery College co‐production settings helped participants rebuild confidence and reimagine their capabilities. One participant shared,

You've been like super disabled and super isolated for so long—and it's like, you can actually contribute—it's really validating (…) and increased confidence.

Participant 11

For some, this renewed confidence came from realising the value in their experiences, as another participant reflected: ‘*I have a contribution to make through who I am’ (Participant 6).* Others described intentionally pushing themselves outside their comfort zone to test their growth. As one participant explained, ‘*I purposely hurl myself out of my comfort zone on a fairly regular basis to see if, you know, maybe I can do this’ (Participant 7).* These reflections highlight how co‐production in Recovery Colleges provided a supportive environment where confidence could be rebuilt through affirmation, challenge and a renewed sense of worth.

Personal growth was also described as achieved by reconnecting with a sense of purpose and meaning from their lived experience. Participants emphasised how co‐produced spaces helped transform past challenges into meaningful contributions. One participant shared, ‘*It is a real reminder to me about just how beautiful things are (…) finding their meaning and purpose again—and reducing those boundaries’ (Participant 1*). Others shared how these experiences allowed them to reframe difficult moments: ‘*it adds value to every moment of struggling that I did—adding value to horrible parts of my life’ (Participant 7)* and ‘*there's value—in all these experiences that traditionally—are overlooked’ (Participant 11).* At the same time, participants discussed the importance of doing this study in an empowering way. As one participant emphasised, ‘*that people with lived and living experience are empowered every step of the way—and authentic and meaningful engagement happens’ (Participant 14).* These reflections highlight how co‐produced environments can foster personal growth by helping individuals find purpose and meaning in their experiences, while ensuring those contributions are honoured and respected.

Finally, participants described how co‐production in Recovery Colleges helped them develop a sense of accomplishment by redefining what success means in the context of mental health and substance use health and recovery. This often meant celebrating quiet victories, such as attending class, speaking up or rediscovering joy that fuelled pride and self‐worth. As one participant shared, ‘*They've learned to recalibrate what success is and success is what we make it’ (Participant 7).* Another emphasised, ‘*There's no force more powerful than a differently abled woman determined to rise’ (Participant 16)*, highlighting the strength and accomplishment from growing through challenges. Participants also expressed accomplishment in stepping into ‘*opportunities in doing things that I never thought I would have done—I'm really proud’ (Participant 14)* and in seeing growth in others: ‘*It's about seeing the impact that Recovery College can have on somebody’ (Participant 10).* These reflections show how co‐produced spaces can support individuals in reclaiming a meaningful sense of achievement.

Participants described co‐produced spaces within the Recovery College setting as fostering personal growth that supports self‐discovery, fosters awareness, rebuilds confidence and helps individuals find purpose and meaning, while developing a sense of accomplishment through their lived/living experience.

## 4 Discussion

This project aimed to understand the impact of co‐production in Recovery College settings on the individuals involved, including those who are co‐producing programming and those who are taking part in co‐produced programming. Results showed that participants found it meaningful to have the space to share lived experience. They also found co‐production to be a stigma‐reducing endeavour that enabled them to develop a sense of belonging, advance their personal well‐being and personal recovery journeys, and achieve personal growth. These themes show that co‐production produces numerous positive impacts across a wide range of personal factors that benefit the individuals and the communities engaged.

Results demonstrated that co‐produced spaces are comfortable, safe, stigma‐free and empowering to the individual, which can help to build a sense of belonging, well‐being and personal recovery. Indeed, Recovery College research has already shown that telling one's story contributes to the recovery journey [26]. The safe and trusting space may produce a sense of ‘common humanity’, which has been put forth as one of the mechanisms of change associated with peer support work in the lived experience framework [27]. This safe and trusting space may also help individuals build their self‐confidence and their trust in others, while potentially repairing losses in trust in the healthcare system, possibly developed during their journey with mental health, substance use health, trauma and disability [28]. Co‐production spaces in Recovery Colleges may also provide a psychologically safe ‘practice space’ where people can learn to listen, learn, practice kindness and respect differences. Indeed, psychologically safe spaces have been shown to support learning in educational spaces [29].

Recovery Colleges were designed to help individuals advance in their recovery through transdiagnostic programming that aids in the development of personal identity [30, 31]. They are non‐clinical, non‐stigmatising spaces where people can develop a sense of belonging [32]. Recovery College research highlights that course participation is a way for people to support their recovery journeys on their own terms. However, as suggested by our results, co‐production may be a secondary means of achieving these goals. The act of co‐producing itself may help people advance in their recovery journeys through altruistic activities of engagement, while also helping to improve the quality of the programming and services produced together [33]. However, for the benefits of co‐production to be realised, a wide range of barriers to co‐production must be addressed, as described in the literature. These include factors such as time constraints, funding requirements, a lack of training, support needed for those co‐producing, precarious working conditions for peers, diversity challenges, and traditional hierarchies in the system [33, 34]. Our study results confirm the literature on the positive impacts of co‐production and did not identify any negative impacts or barriers. A partial explanation for the overwhelmingly positive findings could be that in Recovery Colleges, the people involved in co‐production engage in mutual support, transparency and a true dedication to co‐production while breaking down power dynamics [35], as fits with the very ethos of the Recovery College model. It may be that when barriers are dismantled, positive impacts can be achieved. It is important that the impacts identified herein differ from the substantial impacts also experienced by clinicians in Recovery College settings, which focus more on role definition and power dynamics [36].

### 4.1 Clinical Implications

While Recovery Colleges do not offer clinical interventions, the work does have clinical relevance. Mental health and substance use health service providers in clinical practice should take note of the positive healing impacts of grassroots, real‐world activities like the co‐production of recovery programming. Substantial benefits can be achieved through approaches other than clinical interventions. In mental health and substance use health, recovery programming and healing supports should be holistic and can include unconventional activities [37], which might include co‐production. Indeed, community‐based recovery‐oriented programming has been found to have positive impacts on the mental health of individuals involved [38]. Co‐production could be a cost‐effective and accessible means of helping individuals optimise their mental health, substance use health and personal development, as a complement or alternative to clinical care, which is a relevant area for future research on the cost‐effectiveness of co‐production. It is further possible that these impacts extend beyond Recovery College settings to encompass other areas of co‐production.

### 4.2 Next Steps

The current study was designed as the qualitative preparatory stage to a psychometric development project. We will use the current findings to design a psychometric measurement tool to measure the impact of co‐production on the individuals involved. The themes generated in this project lend themselves to serve as subscales of the future measurement tool. After developing a pilot version of a psychometric measurement tool, we will pilot test it and then submit it to a full study to establish its psychometric properties. The result will be a robust new psychometric assessment tool that measures the impact of co‐production on the individuals involved. This will make it possible to assess some aspects of co‐production that have yet to be thoroughly assessed in the literature [34].

### 4.3 Strengths and Limitations

Through this project, we engaged with people with lived and living experience throughout in the co‐production framework of the CLC Research Subcommittee, which is a considerable strength. Other strengths include a large sample size for a photovoice study, the national scope of the project, and the involvement of a lived experience RA and photographer to guide the work. It was conducted within the framework, values and practices of Recovery Colleges. Individual sessions were available for participants as needed to increase accessibility. However, certain limitations should be kept in mind. Despite efforts to maximise participant diversity, some perspectives may have been missed. Some equity‐deserving subgroups were present in the sample; however, these were in small numbers, and results cannot be derived for specific subgroups of the population. Additional research could be conducted to examine population subgroups of interest. As people self‐selected into the study, they may have had positive opinions about Recovery Colleges and co‐production. We did not ask participants to describe the types of co‐production activities in which they were involved, which can vary substantially across Recovery Colleges [39, 40] and are not always comprehensive [41]. The form of co‐production may affect personal impacts and growth and should be considered in the design of future research. While the virtual nature of the project provided geographic diversity and greater accessibility for many people, it also precluded in‐person activities that might have created more impact for some participants; for example, it may have limited the community development aspect of the photovoice methodology's aims [14]. Future work might consider hybrid means of working. Lastly, while we believe our results shed light on the benefits of co‐production in Recovery College settings more broadly, we recognise that more work would need to be done to examine impacts outside of the Canadian context.

### 4.4 Conclusion

This co‐produced photovoice study on the impacts of co‐production demonstrated a wide range of positive impacts on the individuals involved, across a range of personal factors. It may be that a ripple effect of benefits and impacts takes place in co‐production, because it is about collaborating among a mosaic of voices ebbing towards a positive impact. People achieve personal change through co‐production and the resulting community. The co‐production of services, programming and research should be viewed as a positive and meaningful activity in mental health and substance use health settings, as well as recovery‐oriented programming in Recovery Colleges and beyond.

## Author Contributions

**Lisa D. Hawke:** conceptualization, methodology, formal analysis, visualization, writing – original draft, writing – review and editing, supervision, project administration. **Shelby Mckee:** conceptualization, methodology, formal analysis, writing – original draft, writing – review and editing. **Holly Harris:** conceptualization, methodology, formal analysis, writing – review and editing. **Amy Hsieh:** conceptualization, methodology, formal analysis, writing – review and editing. **James Svoboda:** conceptualization, methodology, formal analysis, writing – review and editing. **Maral Sahaguian:** conceptualization, methodology, formal analysis, writing – review and editing. **Gail Bellissimo:** conceptualization, methodology, formal analysis, writing – review and editing. **Melissa Hiebert:** conceptualization, methodology, formal analysis, writing – review and editing. **Kelly Lawless:** conceptualization, methodology, formal analysis, writing – review and editing. **George James:** conceptualization, methodology, formal analysis, writing – review and editing. **Sean Patenaude:** conceptualization, methodology, writing – review and editing. **Jordana Rovet:** conceptualization, methodology, writing – review and editing. **Sophie Soklaridis:** conceptualization, methodology, formal analysis, writing – review and editing, funding acquisition.

## Ethics Statement

This project was approved by the Research Ethics Board of the Centre for Addiction and Mental Health, #2024‐073.

## Consent

All participants provided electronic, signed informed consent.

## Conflicts of Interest

The authors declare no conflicts of interest.

## Data Availability

The data may be available upon request, with appropriate Research Ethics Board approval.
